# Role of endothelial PDGFB in arterio-venous malformations pathogenesis

**DOI:** 10.1007/s10456-023-09900-w

**Published:** 2023-12-09

**Authors:** Yanzhu Lin, Johannes Gahn, Kuheli Banerjee, Gergana Dobreva, Mahak Singhal, Alexandre Dubrac, Roxana Ola

**Affiliations:** 1 Experimental Pharmacology Mannheim (EPM), European Center for Angioscience (ECAS), Medical Faculty Mannheim, Heidelberg University, Mannheim, Germany; 2 Department of Cardiovascular Genomics and Epigenomics, European Center for Angioscience (ECAS), Medical Faculty Mannheim, Heidelberg University, Mannheim, Germany; 3 German Centre for Cardiovascular Research (DZHK), Heidelberg, Germany; 4 Laboratory of AngioRhythms, European Center for Angioscience (ECAS), Medical Faculty Mannheim, Heidelberg University, Mannheim, Germany; 5 Centre de Recherche, CHU St. Justine, Montreal, QC H3T 1C5 Canada; 6 Département de Pathologie et Biologie Cellulaire, Université de Montréal, Montreal, QC H3T 1J4 Canada

**Keywords:** Pericytes, PDGFB, AVMs, EC migration, Arterial-venous zonation

## Abstract

Arterial-venous malformations (AVMs) are direct connections between arteries and veins without an intervening capillary bed. Either familial inherited or sporadically occurring, localized pericytes (PCs) drop is among the AVMs’ hallmarks. Whether impaired PC coverage triggers AVMs or it is a secondary event is unclear. Here we evaluated the role of the master regulator of PC recruitment, Platelet derived growth factor B (PDGFB) in AVM pathogenesis. Using tamoxifen-inducible deletion of *Pdgfb* in endothelial cells (ECs), we show that disruption of EC *Pdgfb*-mediated PC recruitment and maintenance leads to capillary enlargement and organotypic AVM-like structures. These vascular lesions contain non-proliferative hyperplastic, hypertrophic and miss-oriented capillary ECs with an altered capillary EC fate identity. Mechanistically, we propose that PDGFB maintains capillary EC size and caliber to limit hemodynamic changes, thus restricting expression of Krüppel like factor 4 and activation of Bone morphogenic protein, Transforming growth factor β and NOTCH signaling in ECs. Furthermore, our study emphasizes that inducing or activating PDGFB signaling may be a viable therapeutic approach for treating vascular malformations.

**Supplementary Information:**

The online version contains supplementary material available at 10.1007/s10456-023-09900-w.

## Introduction

Blood vessels form hierarchically branched networks that develop organotypically in a highly unique stereotyped fashion. Once differentiated, maturation and maintenance of this vascular network are provided by stabilizing mural cells (MCs): pericytes (PCs) that envelop small caliber vessels, the capillaries, and vascular smooth muscle cells (vSMCs) covering big caliber vessels, the arteries and veins [1, 2]. Blood flow-induced physiological shear stress is an essential contributor to vessel stabilization in part by regulating the expression of endothelial ligands required for recruitment and maintenance of MCs, e.g. *Pdgfb* (encoding platelet-derived growth factor-B, PDGFB), *Jag1* (encoding Jagged1) or *Tgf-β1* (encoding transforming growth factor β1, TGF-β1) [3, 4]. In turn, the direct contact of MCs with endothelial cells (ECs) allows the MCs to regulate blood flow and the vascular tone within the vascular network [5]. PCs are also important regulators of endothelial angiogenic behavior by limiting VEGF [6] and Angpt2-Tie2 signalling [7], maintaining capillary zonation [8] and suppressing inflammatory responses in the endothelium [9]. Recently, it has been proposed that contractile junctional PCs also dynamically control of blood flow directionality within the capillary network [10].

The recruitment of PCs during angiogenesis is mediated by the angiocrine PDGFB produced by the ECs which paracrine signals through its corresponding receptor PDGF receptor-β (PDGFRβ), exclusively expressed by PCs. Disrupted PDGFB/PDGFRβ signalling pathway results in strongly reduced PC number and various vascular defects, including vascular hyperplasia, microaneurysm, leakage, diabetic retinopathy and impairment in the formation of the blood–brain and blood–retina barrier (BBB, BRB) [7, 9, 11–16].

Impaired PC coverage has also been reported in sporadic human brain arterial-venous malformations (AVMs) with a more pronounced loss in ruptured AVMs [17]. Also, PC dropout is a hallmark of murine Human Hemorrhagic Telangiectasia (HHT)-like AVMs caused by loss of function (LOF) of canonical BMP9 and BMP10 signaling [18–21]. Whether the localized reduction in PC coverage is a consequence of pathological flow, a contributing factor, or a causal determinant of the high flow-AVMs is still not clear. Much less is known about the angiocrine-paracrine signaling pathways whose disruption contributes to PC dysfunction and AVM formation, or whether flow-induced PDGFB activation is implicated.

NOTCH signalling acting upstream of PDGFB, also promotes PC recruitment and adhesion to microvessels during vascular morphogenesis. Loss of canonical NOTCH in perivascular cells [22] or gain of NOTCH signaling in ECs [23] has been associated with PC reduction and AVM pathogenesis. A similar phenotype was observed when *Srf* (Serum response factor) transcription factor, acting downstream of PDGFB-PDGFRβ, is depleted from MCs [24].

In the present study, we evaluated the role of PDGFB signaling-mediated PC recruitment and maintenance in AVM pathogenesis. Our data show that disruption of endothelial *Pdgfb* resulting in PC loss in developing vessels leads to capillary enlargement giving rise to organotypic arterio-venous (AV) shunting containing non-proliferative hyperplastic, hypertrophic and miss-oriented ECs with an altered AV capillary zonation. Mechanistically, loss of *Pdgfb* is associated with an increase in Krüppel like factor 4 (KLF4) expression-meditated excessive Bone morphogenic protein (BMP), TGF-β and NOTCH activation in ECs. Collectively, the data here suggest that PDGFB-mediated PC recruitment and maintenance on developing vessels restrict capillary EC size and caliber to prevent hemodynamic changes and AV shunting. Furthermore, our study identifies novel targets with the potential to prevent vascular lesions.

## Results

### PDGFB-mediated PC recruitment regulates retinal angiogenesis and capillary size

**Fig. 1:**
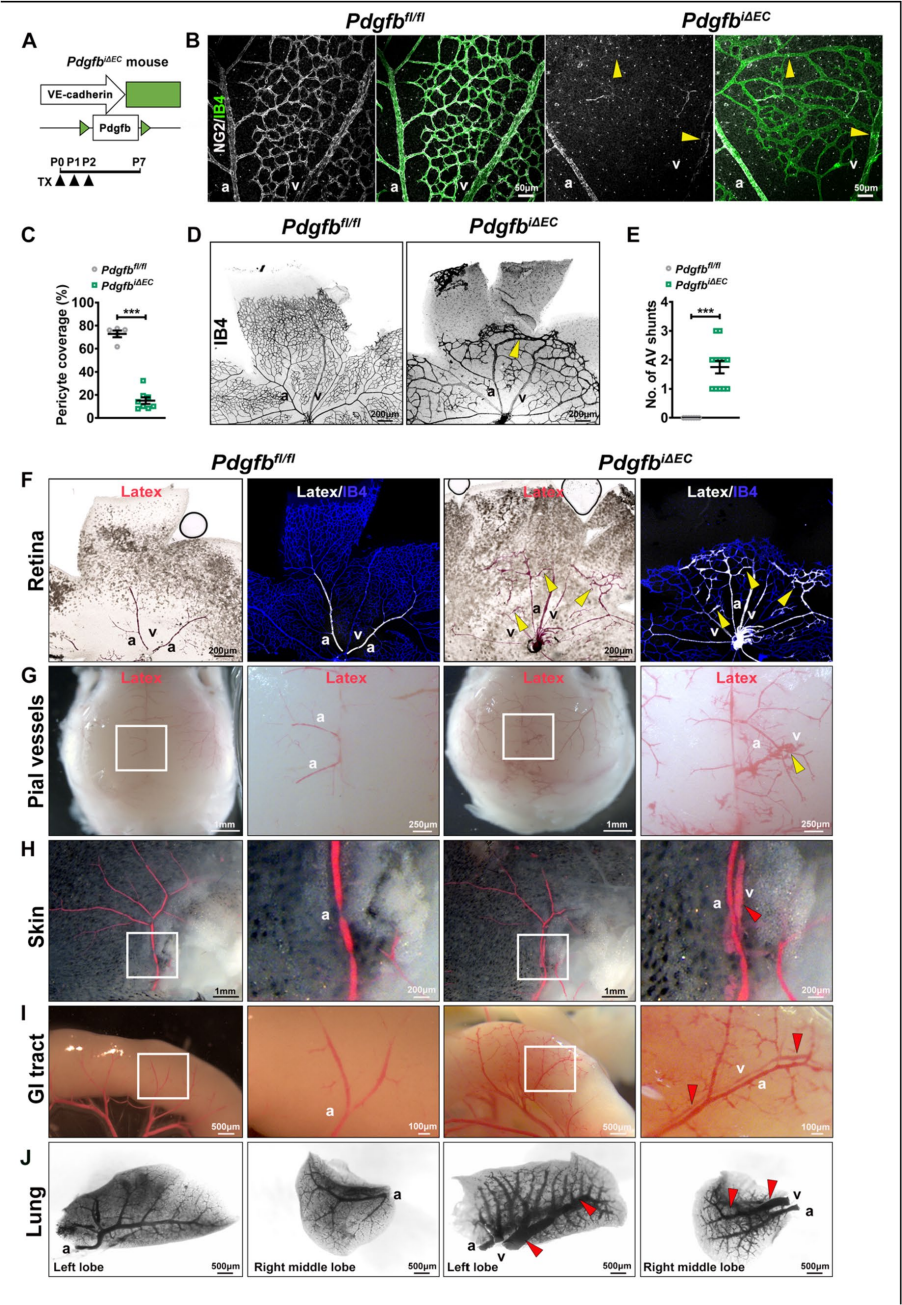
Loss of EC *Pdgfb*-mediated PC recruitment results in capillary enlargement and AV shunting. **A** Schematic of the experimental strategy used to delete *Pdgfb* in ECs (P0–P7). Arrowheads indicate i.p injection of 100 μg TX at P0-P2 in *Pdgfb*^*fl/fl*^ and *Pdgfb*^*i∆EC*^. **B** Labeling for NG2 (white) and IB4 (green) of P7 *Pdgfb*^*fl/fl*^ and *Pdgfb*^i∆EC^ retinas. Yellow arrowheads point to loss of PC coverage in the capillaries and veins. **C** Quantification of the PC coverage in *Pdgfb*^*fl/fl*^ and *Pdgfb*^*iΔEC*^ (*Pdgfb*^*fl/fl*^ n = 5, *Pdgfb*^*i∆EC*^ n = 8, unpaired 2-tailed *t* test). **D** Labeling with IB4 (negative images) of *Pdgfb*^*fl/fl*^ and *Pdgfb*^*i∆EC*^ P7 retinas. **E** Quantification of the number of AV shunts in *Pdgfb*^*fl/fl*^ and *Pdgfb*^*iΔEC*^ P7 retinas (*Pdgfb*^*fl/fl*^ n = 7, *Pdgfb*^*i∆EC*^ n = 12, Mann–Whitney *U* test). **F**–**J** Images of P7 retinas (**F**), pial vessels (**G**), skin (**H**), GI tracts—small intestine (**I**) and lungs (negative images) (**J**) in *Pdgfb*^*fl/fl*^ and *Pdgfb*^*iΔEC*^ mice perfused with latex red-dye. Perfused retinas in **F** were subsequently stained with IB4 (blue). Yellow arrowheads in **D**, **F**, **G** mark AV shunts. Red arrowheads in **H**, **I** and** J** mark latex-perfused veins in the skin, GI tracts and lungs. ***P < 0.001. *a* artery, *v* vein

To address the role of PDGFB-mediated PC recruitment in AVM pathogenesis, we depleted *Pdgfb* specifically in ECs by crossing *Pdgfb*^*fl/fl*^ to the tamoxifen (TX) inducible Cdh5-CreERT2 mouse line to create *Pdgfb*^*iΔEC*^ (Fig. 1A). *Pdgfb* gene depletion was induced in newborns via intraperitoneal (i.p) TX administration at postnatal days (P0–P2 and retinal vasculature was analyzed at P7. Efficient gene depletion was confirmed by qPCR and western blot (WB) in mouse lung ECs (mLECs) isolated from TX induced P7 pups (Suppl. Fig. 1a, b). Loss of *Pdgfb* resulted in no significant changes in the body weight compared to control neonates (*Pdgfb*^*fl/fl*^) (Suppl. Fig. 1c). Labeling P7 *Pdgfb*^*fl/fl*^* and Pdgfb*^*iΔEC*^ retinas for myocardial neuron-glial antigen 2 (NG2), a MC marker, and Isolectin B4 (IB4) to detect the ECs, revealed severely impaired PC coverage of the developing vessels (Fig. 1B, quantified in C) with multiple vascular defects: decrease in vascular area and outgrowth, loss of side branches, and vessel dilation with the highest impact within the capillaries and a significant decline in the morphological altered tip cell number with fewer and shorter filopodia (Fig. 1D; Suppl. Fig. 1d–f), thus confirming previous findings [7, 25, 26].

Interestingly, in addition to the previously described phenotypes, loss of *Pdgfb* led to direct connections between arteries and veins at the vascular front, on average 1.7 connections per retina (arrowhead in Fig. 1D, quantified in E). To confirm whether these direct connections between arteries and veins are indeed AV shunts, we injected latex red-dye into the left side of the neonatal P7 hearts [20]. Due to its size, the latex cannot pass the capillary bed and remains confined to arterial branches. Yet, in *Pdgfb*^*iΔEC*^ retinas, latex was present in the enlarged capillaries and into the retinal veins, suggesting direct AV connections between arteries and veins upon *Pdgfb* LOF (Fig. 1F). To identify if *Pdgfb* loss leads to AV shunting in other organs, we analyzed other vascular beds following intracardial latex injection. Interestingly, we found AV shunting also in the pial vessels (Fig. 1G) and latex perfused veins in the skin of the scalp, gastrointestinal (GI) tract (small intestine) and lungs of mutant neonates, whereas no perfused veins were seen in control neonates (Fig. 1H–J). These findings may indicate either capillary enlargement in visceral organs, or presence of direct AV shunting at a downstream or upstream location from the visualized vascular field. Thus, PDGFB-mediated PC recruitment regulates retinal angiogenesis and capillary size.

### Endothelial PDGFB is required for PC maintenance to control the capillary size

**Fig. 2:**
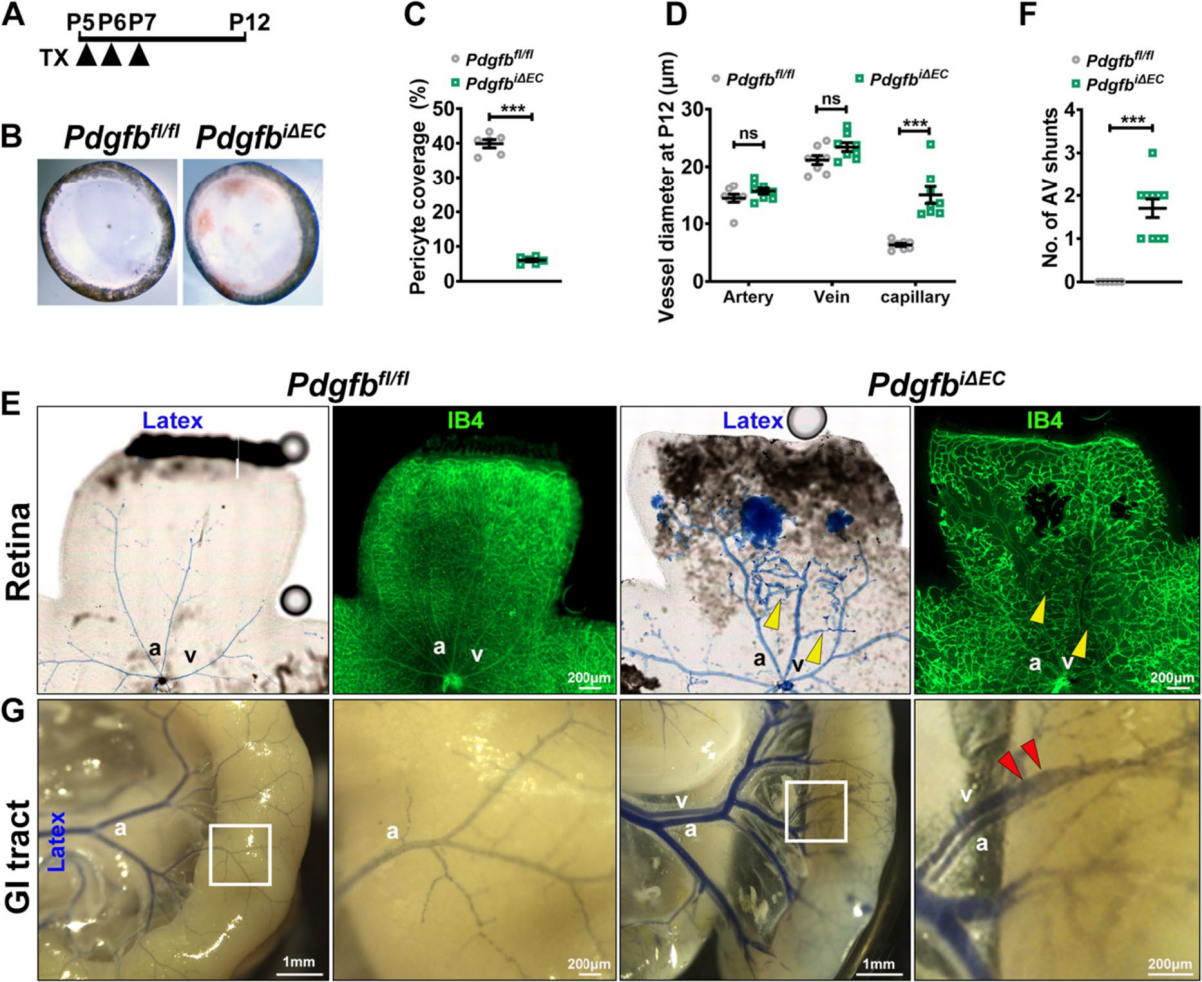
Endothelial PDGFB is required for PC maintenance to restrict the capillary size. **A** Schematic of TX administration in postnatal *Pdgfb*^*fl/fl*^ and *Pdgfb*^*iΔEC*^ mice at P5-P7 and analysis at P12. **B** Representative images of hemorrhagic whole retinas isolated from *Pdgfb*^*fl/fl*^ and *Pdgfb*^*iΔEC*^ P12 neonates. **C** Quantification of PC coverage in P12 retinas (*Pdgfb*^*fl/fl*^ n = 6, *Pdgfb*^*i∆EC*^ n = 6, unpaired 2-tailed *t* test with Welch’s correction). **D** Quantification of the retinal vessel diameter in *Pdgfb*^*fl/fl*^ and *Pdgfb*^*iΔEC*^ retinas (*Pdgfb*^*fl/fl*^ n = 8, *Pdgfb*^*i∆EC*^ n = 8, Mann–Whitney *U* test). **E** Images of *Pdgfb*^*fl/fl*^ and *Pdgfb*^*iΔEC*^ P12 retinas stained for IB4 (green) latex perfused (blue). **F** Quantification of the number of AV shunts in P12 retinas (*Pdgfb*^*fl/fl*^ n = 6, *Pdgfb*^*i∆EC*^ n = 10, Mann–Whitney *U* test). **G** Images of P12 GI tracts latex perfused. Yellow arrowheads in **E** mark latex perfused retinal AV shunts. Red arrowheads in **G** mark the latex-perfused veins in the GI tract. *ns* non-significant, ***P < 0.001. *a* artery, *v* vein

To further understand if PDGFB-mediated maintenance of PCs on developing endothelium is required to control capillary size, we depleted *Pdgfb* at P5–P7 and analyzed retinas at P12 (Fig. 2A). Interestingly, loss of *Pdgfb* at this stage led to increased retinal hemorrhage (Fig. 2B), and impaired PC coverage as quantified by the NG2 + /IB4 + vascular area (Fig. 2C). By measuring vessel diameter at P12, we found a significantly altered capillary enlargement, while the diameter of veins or arteries remained unaffected (Fig. 2D). Retinal AV shunting occurred with an average of 1.7 connections per retina as shown by IB4 + endothelium and latex positive capillaries and veins (Fig. 2E, quantified in F). Surprisingly, at this stage, the AVM-like structures formed closer to the optic nerve, in higher flow regions (arrowheads in Fig. 2E), suggesting that blood flow participates in AV shunt formation upon *Pdgfb* depletion. Latex dye-positive veins were also observed in the GI tract (Fig. 2G). These findings argue that PDGFB is indispensable for the maintenance of PCs on developing endothelium to restrict capillary size and protect against retinal AV shunting.

### Endothelial PDGFB regulates capillary EC size and polarity

The *Pdgfb* LOF phenotype was extensively described [7, 26], yet AV shunt formation hasn’t been reported so far. Therefore, we focused on characterizing the cellular hallmarks of AVM-like structures in P7 *Pdgfb*^*iΔEC*^ retinas. Upon staining retinas for alpha smooth muscle actin (αSMA), we found a strong reduction in arterial vascular smooth muscle cells (vSMC) coverage, with scattered αSMA + immunostaining in capillaries and more abundant in veins but no ectopic﻿ αSMA ﻿﻿within the AV shunt capillaries (Suppl Fig. 2a, quantified in b). Labelling retinas for IB4 and ICAM2, a cell adhesion molecule located at the apical EC surface further confirmed an increase in capillaries’ lumen (Suppl Fig. 2c, quantified in d). Staining for Collagen IV (ColIV) that labels the basal surface identified a higher number of empty sleeves (IB4-/ColIV +) (Suppl Fig. 2e, quantified in f), indicating excessive vascular pruning events in *Pdgfb*^*iΔEC*^ retinas.

We next reasoned that the expansion of capillary diameter is due to, either an increase in the size of capillary ECs or due to proliferating ECs clustering within capillaries. Another possibility is that perhaps disrupted EC migration against the bloodstream contributes to lumen enlargement, as capillary regression in developing vascular plexus is a cell migration–driven process from low towards higher flow regions [27].

**Fig. 3:**
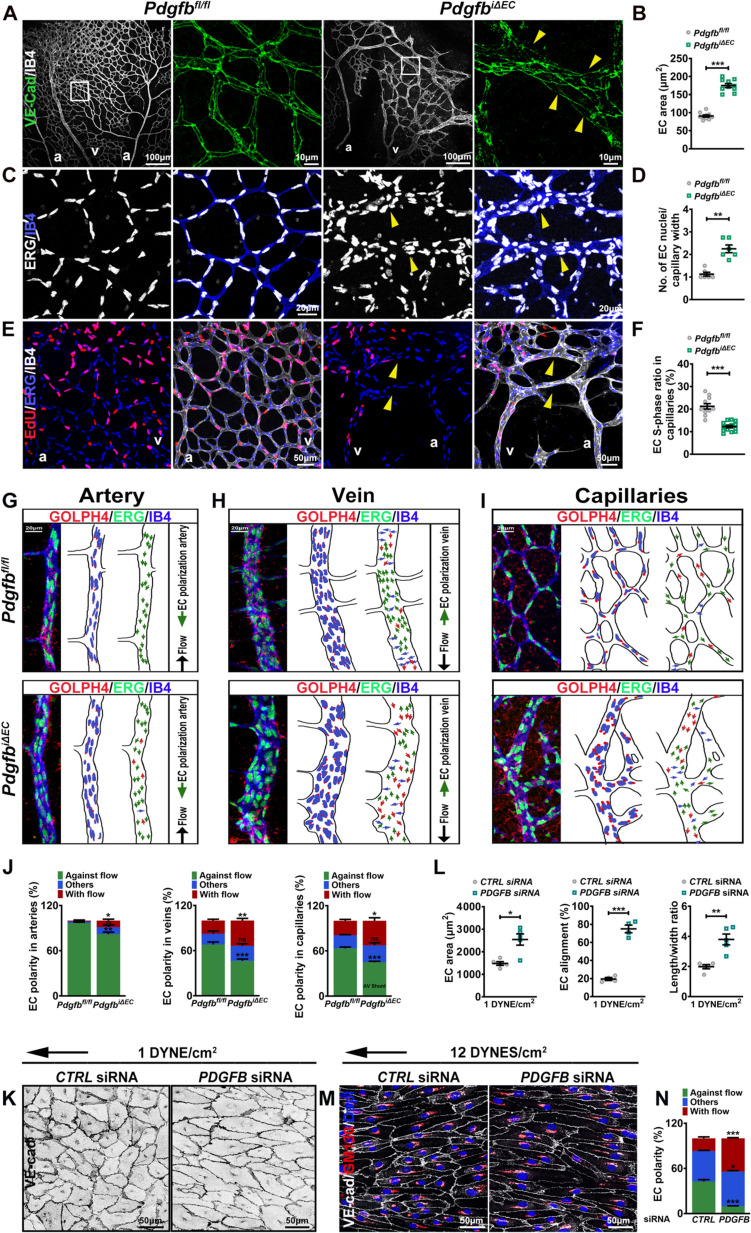
EC PDGFB maintains capillary EC size and EC polarity against the bloodstream. **A**, **C**, **E**, **G–I** Co-immunolabeling for VE-Cadherin (green) and IB4 (white) (**A**), ERG (white) and IB4 (blue) (**C**), EdU (red), ERG (blue) and IB4 (white) after 4 h of EdU incorporation (**E**), and for ERG (green), IB4 (blue) and GM130 (Golgi marker-red) in arteries, veins and capillaries (**G–I**) of P7 *Pdgfb*^*fl/fl*^ and *Pdgfb*^*iΔEC*^ retinas. **G–I** Right side, panels illustrating EC polarization based on the position of the Golgi apparatus in relation to the nucleus in the direction of migration (green arrows). **B**, **D**, **F** Quantification of the EC size (*Pdgfb*^*fl/fl*^ n = 10, *Pdgfb*^*iΔEC*^ n = 10, unpaired 2-tailed *t* test) (**B**), of the number of EC nuclei per capillary width (*Pdgfb*^*fl/fl*^ n = 6, *Pdgfb*^*iΔEC*^ n = 6, Mann–Whitney *U* test) (**D**) and of EdU + ERG + /ERG + ECs (*Pdgfb*^*fl/fl*^ n = 11, *Pdgfb*^*iΔEC*^ n = 14, unpaired 2-tailed *t* test) (**F**). **J** Quantification of EC polarization: against the direction of flow (green arrows), towards the direction of flow (red arrows) and non-oriented (blue arrows) in arteries (**G**), veins (**H**) and capillaries (**I**) from P7 TX induced retinas from the indicated genotypes (*Pdgfb*^*fl/fl*^ n = 4/5, *Pdgfb*^*iΔEC*^ n = 6/8, unpaired 2-tailed *t* test). Yellow arrowheads in **A** point towards capillary ECs with loss of VE-cadherin at the cell–cell junctions, in **C** towards the enlarged capillaries containing more than one EC and in** E** towards the EdU- ECs within the AV shunt. **K** VE-Cadherin staining (negative images) of *CTRL* and *PDGFB* siRNA HUVECs subject to 1 DYNE/cm^2^. **L** Quantification of EC area, EC alignment and length/width ratio in *CTRL* and *PDGFB* siRNA HUVECs subject to 1 DYNE/cm^2^ (n = 4/5 average of 3 images (70–140 cells/image) per 3 independent experiments/group, unpaired 2-tailed *t* test). **M** Labelling of *CTRL* and *PDGFB* siRNA HUVECs subject to 12 DYNES/cm^2^ for GM130 (Golgi apparatus), VE-Cadherin and DAPI (nuclei) (**N**) quantification of EC orientation against the direction of flow, with the flow and others (non-oriented). *ns* non-significant, *P < 0.05, **P < 0.01, ***P < 0.001. *a* artery, *v* vein

Staining retinas for VE-cadherin identified larger capillary single ECs and loss of VE-cadherin at cell–cell junctions (Fig. 3A, quantified in B), consistent with the increased hemorrhage observed in P12 mutant retinas (in Fig. 2B). Furthermore, labeling for the endothelial nuclear ERG1,2,3 transcription factor (ERG) [28] and IB4 identified an increase in the number of capillary EC nuclei per capillary width (Fig. 3C, quantified in D). In control retinas, capillaries contained a single EC, whereas in *Pdgfb*^*iΔEC*^ retinas, capillaries contained two to three EC nuclei (Fig. 3C, quantified in D).

To assess if EC proliferation leads to capillary expansion, we further quantified the proportion of ECs in S-phase (EdU + ERG + /ERG +) upon 5-ethynyl-2′-deoxyuridine (EdU) incorporation in capillary ECs at the vascular retinal front in *Pdgfb*^*fl/fl*^ and *Pdgfb*^*iΔEC*^ neonates. We found an overall decrease in the total number of ERG + ECs with fewer EdU + ERG + cells within the AVM-like structures (Fig. 3E, quantified in F), suggesting that capillary enhancement does not involve proliferation. Furthermore, labeling retinas for P21 encoded by *CDKN1A* (cyclin dependent kinase inhibitor 1A), an indicator of cell cycle arrest [29], showed an increase in the number of arrested ECs (P21 + ERG + cells) within the dilated capillaries (Suppl. Fig. 2g, quantified in h).

Failure of ECs to orient and migrate against the bloodstream was proposed to trigger AVM formation upon depletion of BMP9/10 signaling receptors, *Alk1* and *Eng* in ECs [30, 31]. This physiological process is crucial for vessel regression-mediated vascular remodeling and artery formation [27, 32–34]. To identify if defective flow-mediated EC migration contributes to capillary enlargement and AV shunting, we labelled *Pdgfb*^*fl/fl*^ and *Pdgfb*^*iΔEC*^ retinas for Golph4 (to detect the Golgi apparatus), ERG and IB4. Quantifying the Golgi position in relation to the nucleus and flow direction, we found a significant altered orientation of *Pdgfb* mutant ECs against the bloodstream in arteries, with a more pronounced EC polarization with the flow in veins and capillary ECs engaged in AV shunts (Fig. 3G–I, quantified in J). Taken together, these results suggest that capillary enlargement does not involve proliferation but instead an increase in single EC size and disrupted EC migration against the bloodstream.

As capillary enlargement leads to changes in blood flow or fluid shear stress (FSS), we further assessed the cellular effects of *PDGFB* depletion (using the siRNA strategy) in HUVECs subject to low fluid shear stress (L-FSS) (1 DYNE/cm^2^). Remarkably, *PDGFB* depleted HUVECs under 1 DYNE/cm^2^ were larger, more elongated and aligned parallel to the direction of flow, whereas no significant changes were observed in *CTRL* siRNA HUVECs (Fig. 3K, quantified in L). We further addressed the effect of *PDGFB* loss on EC orientation in response to physiological flow (12 DYNES/cm^2^). Labelled HUVECs for GM130 (for Golgi apparatus), DAPI (for nucleus) and VE Cadherin showed dysregulated migration against the flow direction, with more ECs polarized with the flow upon *PDGFB* loss (Fig. 3M, quantified in N). Thus, *PDGFB* depletion amplifies morphological responses to FSS e.g. EC size, elongation and alignment and leads to disrupted EC orientation against the FSS direction.

### Loss of Pdgfb leads to an altered capillary arterial-venous zonation

**Fig. 4:**
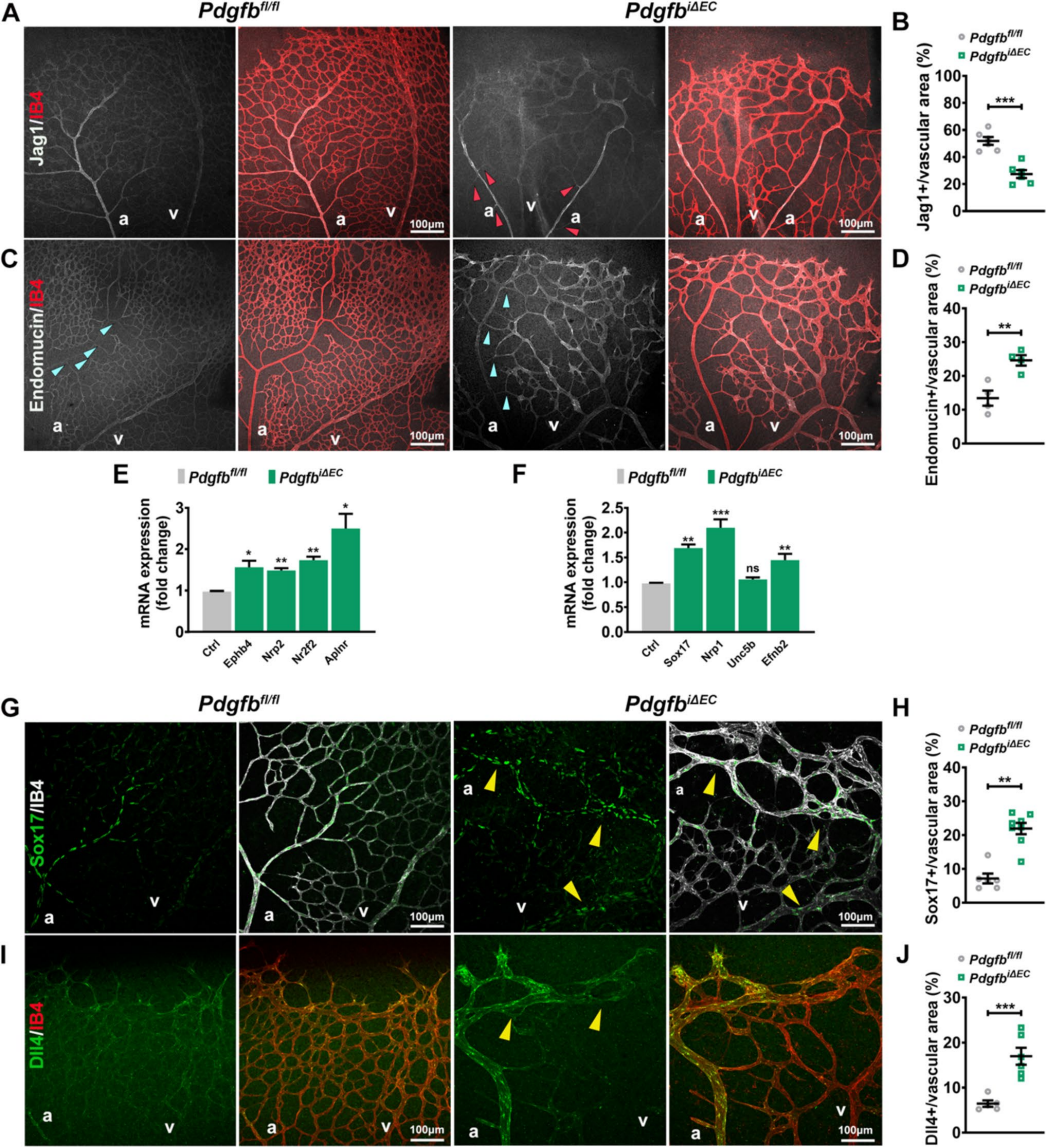
Endothelial PDGFB maintains capillary arterial-venous zonation. **A**, **C**, **G**, **I** Immunostaining of P7 *Pdgfb*^*fl/fl*^ and *Pdgfb*^*iΔEC*^ retinas for Jag1 (white) and IB4 (red) (**A**), Endomucin (white) and IB4 (red) (**C**), Sox17 (green) and IB4 (white) (**G**), Dll4 (green) and IB4 (red) (**I**). **B**, **D**, **H**, **J** Quantification of the labelling intensity of Jag1 (*Pdgfb*^*fl/fl*^ n = 6, *Pdgfb*^*iΔEC*^ n = 6, unpaired 2-tailed *t* test) (**B**), Endomucin (*Pdgfb*^*fl/fl*^ n = 4, *Pdgfb*^*iΔEC*^ n = 4, unpaired 2-tailed *t* test) (**D**), Sox17 (*Pdgfb*^*fl/fl*^ n = 6, *Pdgfb*^*iΔEC*^ n = 8, Mann–Whitney *U* test) (**H**) and Dll4 (*Pdgfb*^*fl/fl*^ n = 5, *Pdgfb*^*iΔEC*^ n = 6, unpaired 2-tailed *t* test) (**J**), per vascular area in P7 *Pdgfb*^*fl/fl*^ and *Pdgfb*^*iΔEC*^ retinas. **E**, **F** qPCR for indicated venous markers (*Pdgfb*^*fl/fl*^ n = 4, *Pdgfb*^*iΔEC*^ n = 4, unpaired 2-tailed *t* test with Welch’s correction) (**E**), and arterial markers (*Pdgfb*^*fl/fl*^ n = 4, *Pdgfb*^*iΔEC*^ n = 4, unpaired 2-tailed *t* test with Welch’s correction) (**F**) in mLECs from *Pdgfb*^*fl/fl*^ and *Pdgfb*^*iΔEC*^ mice. *P < 0.05, **P < 0.01, ***P < 0.001. *a* artery, *v* vein

Previous analysis of single-cell RNAseq datasets identified defective capillary zonation with venous skewing in the brain microvasculature of *Pdgfb* hypomorphs (*Pdgfb*^ret/ret^), pointing towards the role of PCs in maintaining capillary arterio-venous differentiation [35]. To identify if an altered arterial-venous zonation occurs in AVM-like structures in the retina, we immunolabeled control and *Pdgfb*^*iΔEC*^ retinas for several arterial and venous markers at P7. While JAG1, a ligand of NOTCH1 marks predominantly the arteries and arterioles in control retinas [36], in *Pdgfb* LOF retinas, JAG1 expression was retained in arteries but decreased in the arterioles (red arrows in Fig. 4A, quantified in B). In contrast, Endomucin, a transmembrane sialomucin expressed exclusively on the surface of capillary and venous ECs [37, 38], displayed an opposite pattern in *Pdgfb*^*iΔEC*^ vasculature, its expression was upregulated in all capillary ECs expanding also into arterioles (Fig. 4C, quantified in D). These results suggest gain of venous identity and loss of arterial identity. To confirm venous skewing upon *Pdgfb* loss, we performed qPCR on isolated mLECs and observed upregulation of the venous markers, e.g. *EphB4*, *Nrp2*, *Nr2f2* and *Aplnr* (Fig. 4E). Surprisingly though, we identified an upregulation of the arterial markers (*Sox17*, *Nrp1*, *Efnb2*) as well (Fig. 4F).

To dig further into possible gain of arterial identity, we labeled retinas for arterial markers: Sox17 (SRY-box transcription factor 17) which plays a crucial role in arterial identity maintenance [39] and Notch ligand Delta-like 4 (Dll4), another NOTCH1 ligand. Interestingly, upon *Pdgfb* loss, Sox17 expression extended exclusively throughout the AV shunt from the artery towards the vein, with some Sox17 positive ECs present within the vein (arrowheads in Fig. 4G, quantified in H). A similar pattern was observed also for Dll4 (arrowheads in Fig. 4I, quantified in J).

Thus, our results together argue that the AV shunting upon *Pdgfb* loss involve gain of arterial identity with more arterial ECs migrated from the artery into the veins and point towards an arterial origin for the AVMs-like structures.

### AVM-like structures upon endothelial Pdgfb loss have a non-arterial origin

**Fig. 5:**
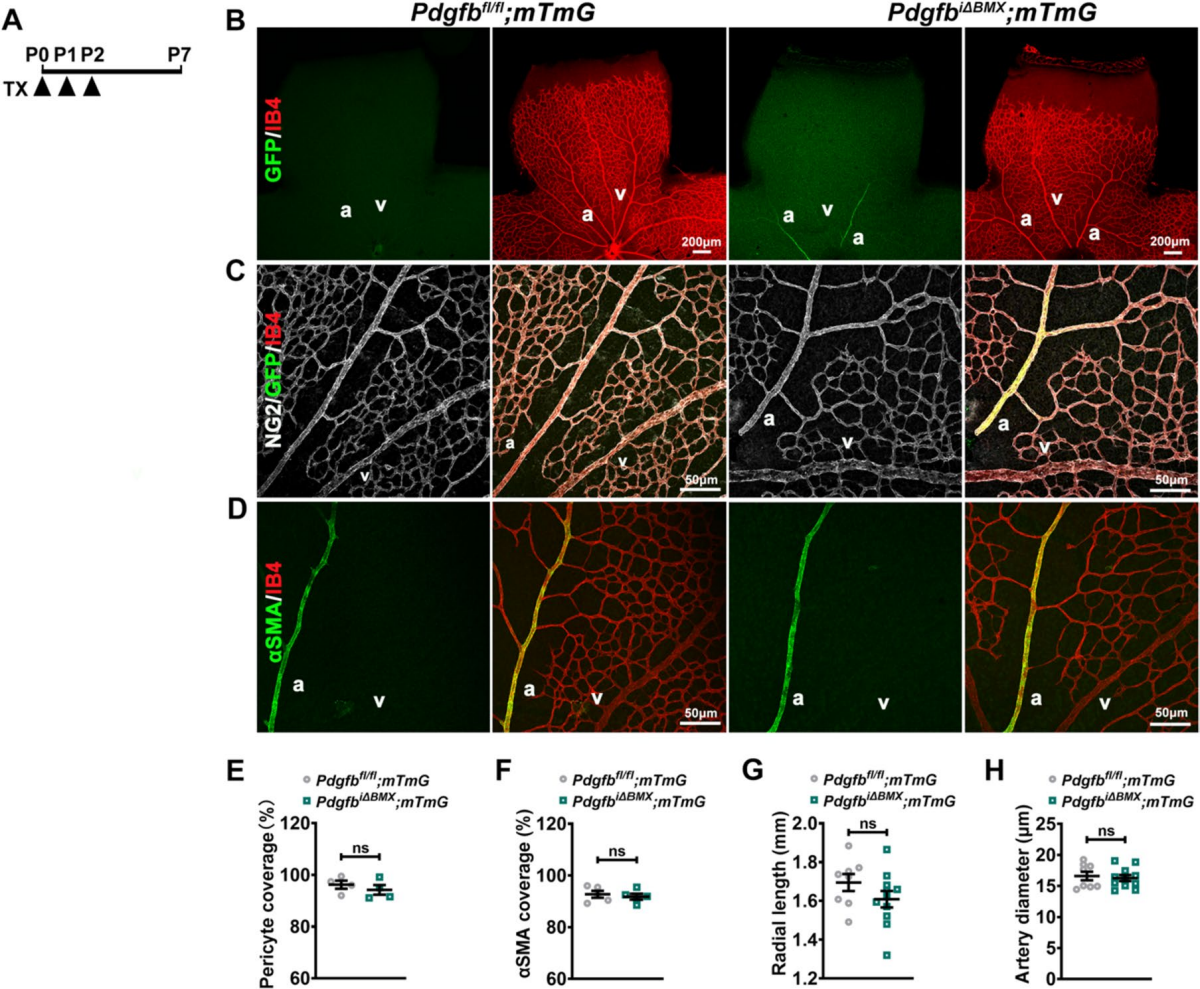
AVM-like structures upon *Pdgfb* loss have a non-arterial origin. **A** Schematic representation of the experimental strategy to delete *Pdgfb* (P0–P7) in arterial ECs using the Bmx-Cre;mTmG reporter mice. Arrowheads indicate i.p injection of 100 μg TX at P0-P2 in *Pdgfb*^*fl/fl*^;*mTmG* and *Pdgfb*^*i∆BMX*^;*mTmG*. **B**, **C**, **D** Representative images of P7 TX induced *Pdgfb*^*fl/fl*^;*mTmG* and *Pdgfb*^*i∆BMX*^;*mTmG* retinas labelled for GFP (green) and IB4 (red) (**B**), NG2 (white), GFP (green) and IB4 (red) (**C**), αSMA (green) and IB4 (red) (**D**). **E**–**H**) Quantification of  PC coverage (%) (*Pdgfb*^*fl/fl*^ n = 4, *Pdgfb*^*i∆EC*^ n = 4, unpaired 2-tailed *t* test) (**E**), αSMA coverage (%) (*Pdgfb*^*fl/fl*^ n = 5, *Pdgfb*^*i∆EC*^ n = 5, unpaired 2-tailed *t* test) (**F**), radial length (*Pdgfb*^*fl/fl*^ n = 8, *Pdgfb*^*i∆EC*^ n = 11, unpaired 2-tailed *t* test) (**G**) and artery diameter (*Pdgfb*^*fl/fl*^ n = 8, *Pdgfb*^*i∆EC*^ n = 11, unpaired 2-tailed *t* test) (**H**) in P7 *Pdgfb*^*fl/fl*^;*mTmG* and *Pdgfb*^*i∆BMX*^;*mTmG*. *ns* non-significant, *a* artery, *v* vein

To test the hypothesis that AVMs-like structures upon *Pdgfb* LOF are of arterial origin, we intercrossed *Pdgfb*^*fl/fl*^ mice to an artery-specific BMX non-receptor tyrosine kinase (BMX) mouse line and *mTmG* reporter mice to lineage-trace green fluorescent protein (GFP) in *Bmx* + and *Pdgfb* depleted ECs (*Pdgfb*^*iΔBMX*^*;mTmG*). Tx was injected at P0-P2, and retinas were analyzed at P7 (Fig. 5A). GFP was detected in arteries and some arterioli of *Pdgfb*^*iΔBMX*^*;mTmG* retinas confirming Cre-mediated recombination specifically in arterial ECs (Fig. 5B). We found that *Pdgfb*^*iΔBMX*^*;mTmG* mutants displayed no retinal AVMs (Fig. 5B), and loss of *Pdgfb* specifically in arterial ECs led to a non-significant loss of PCs (Fig. 5C, quantified in E) or αSMA (Fig. 5D, quantified in F) with no obvious vascular defects (Fig. 5G, H). These results imply that PDGFB is dispensable for artery maintenance and in the same time suggest a non-arterial origin for the AVM-like upon *Pdgfb* loss.

### PDGFB-mediated PC recruitment is associated with TGF-β/BMP and NOTCH signaling activation

As we identified a mixed arterial-venous zonation within the AV shunts, we next focused on signaling pathways regulating arterial-venous identity, whose disruption may contribute to AV shunting in *Pdgfb* LOF mice. In addition to a well-described role of NOTCH signaling in maintaining arterial identity [33, 40, 41], more recently it has been proposed that TGFβ and BMP signaling pathways regulate arterial and venous identity, respectively [42].

**Fig. 6:**
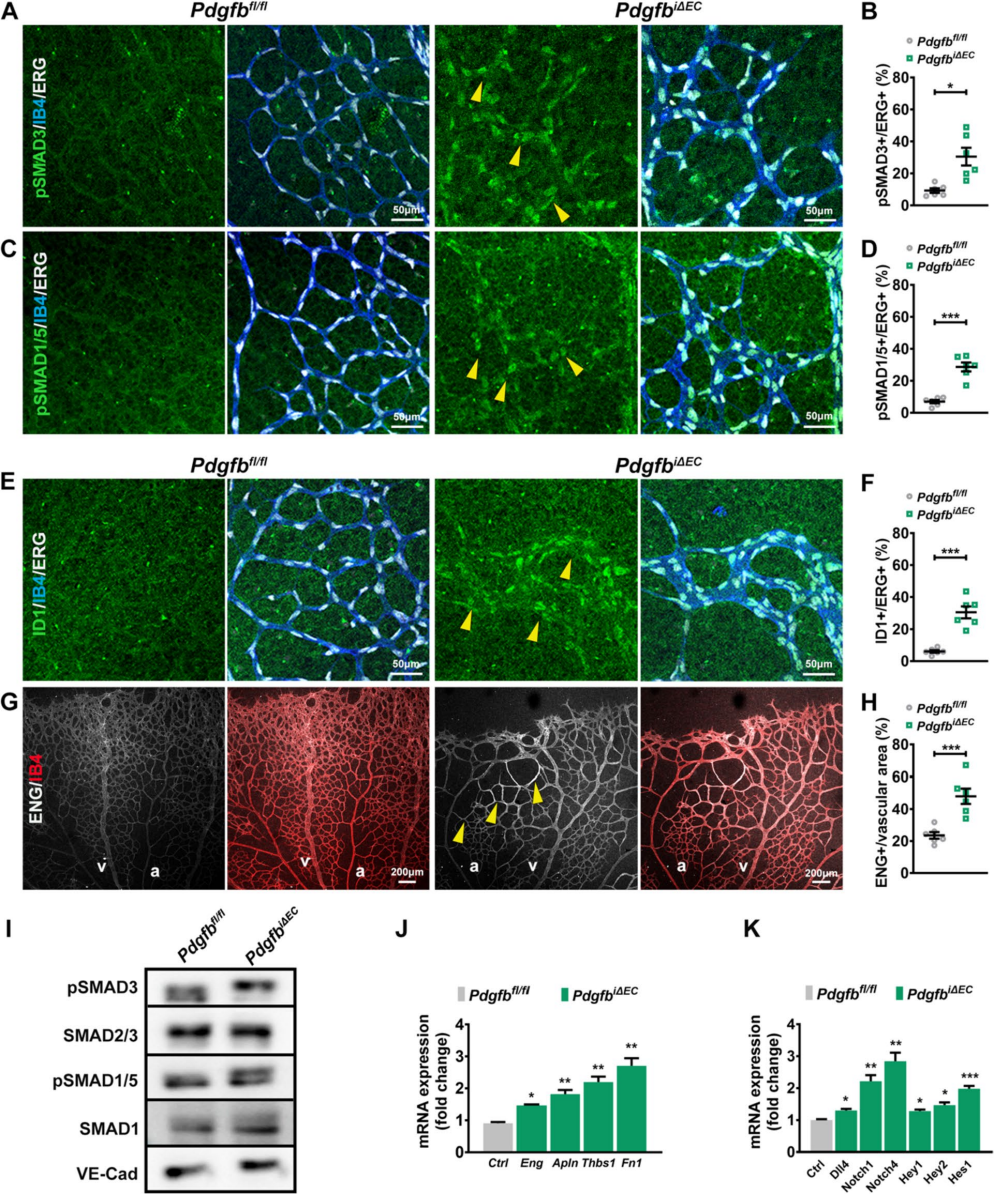
Loss of endothelial *Pdgfb* is associated with activation of TGFβ/BMP and NOTCH signaling pathways. **A**, **C**, **E**, **G** Co-labelling of P7 *Pdgfb*^*fl/fl*^ and *Pdgfb*^*iΔEC*^ retinas with IB4 (blue), Erg (white) and pSMAD3 (green) (**A**), IB4 (blue), ERG (white) and pSMAD1/5 (green) (**C**), IB4 (blue), ERG (white) and ID1 (green) (**E**) ENG (white) and IB4 (red) (**G**). **B**, **D**, **F**, **H** Quantification of the labelling intensity per vascular area (%) of pSMAD3 (**B**), pSMAD1/5 (**D**), ID1 (**F**) and of ENG (**H**) in P7 retinas *Pdgfb*^*fl/fl*^ and *Pdgfb*^*iΔEC*^ retinas (*Pdgfb*^*fl/fl*^ n = 6, *Pdgfb*^*i∆EC*^ n = 6, unpaired 2-tailed *t* test with Welch’s correction). Yellow arrowheads point to upregulated proteins in dilated capillaries upon *Pdgfb* LOF. **I** WB of *Pdgfb*^*fl/fl*^ and *Pdgfb*^*iΔEC*^ mLECs for activated pSMAD1/5 and pSMAD3 and total SMAD1, SMAD2/3 and VE-Cadherin as a loading control. **J**, **K** qPCR for TGFβ/BMP (**J**) and NOTCH target genes (*Pdgfb*^*fl/fl*^ n = 4, *Pdgfb*^*i∆EC*^ n = 4, unpaired 2-tailed *t* test with Welch’s correction) (**K**). *P < 0.05, **P < 0.01, ***P < 0.001. *a* artery, *v* vein

As a readout for TGFβ/BMP pathway activation, we labelled *Pdgfb*^*fl/fl*^* and Pdgfb*^*iΔEC*^ retinas for phosphorylated canonical SMADs. Surprisingly, we identified abundant expression in ECs of both activated canonical SMADs, the pSMAD3 (Fig. 6A, quantified in B) and pSMAD1/5 (Fig. 6C, quantified in D) at the highest intensity within the enlarged capillaries engaged in AVMs-like structures. Furthermore, activation of the canonical SMADs was accompanied by upregulation of TGFβ/BMP target genes [43–45], ID1 (Fig. 6E, quantified in F) and ENG (Fig. 6G, quantified in H). To validate these results, we performed WB for the activated SMADs (Fig. 6I) and qPCR analysis for the TGFβ/BMP target genes in mLECs isolated from TX induced P7 *Pdgfb*^*fl/fl*^ and *Pdgfb*^*iΔEC*^ mice (Fig. 6J). We confirmed increased phosphorylation in SMAD3 and SMAD1/5 (Fig. 6I) and upregulation of TGFβ/BMP downstream target genes: *Eng*, *Thbs1* (Thrombospondin1) and *Fn1* (Fibronectin), thus validating activation of TGFβ/BMP signaling pathways upon loss of *Pdgfb* in ECs. Interestingly, opposite as expected, we identified upregulation of *Apln* (Apelin) (Fig. 6J), a known suppressed BMP target gene [46].

As gain of NOTCH signaling in ECs also leads to AV shunting, and we detected increased Dll4 within the enlarged capillary ECs, we performed qPCR for NOTCH transducers and transcriptional effectors and identified an increase in the expression of *Notch1* and *Notch4 *receptors and upregulated transcription factors *Hey1*, *Hey2* and *Hes1* (Fig. 6K), indicating, additionally, EC NOTCH activation upon *Pdgfb* loss.

These results imply that loss of arterial-venous zonation upon *Pdgfb* depletion is associated with increased TGFβ/BMP and Notch activation in ECs.

### Capillary enlargement results in pathological flow-mediated KLF4 upregulation

Physiological blood flow-induced FSS maintains vascular stability by mediating MC recruitment [3, 4] and promoting EC elongation, alignment [47] and arterial cell fate [48, 49]. In turn, MCs regulate the blood flow and vascular tone [5], but also the blood flow directionality [10].

**Fig. 7:**
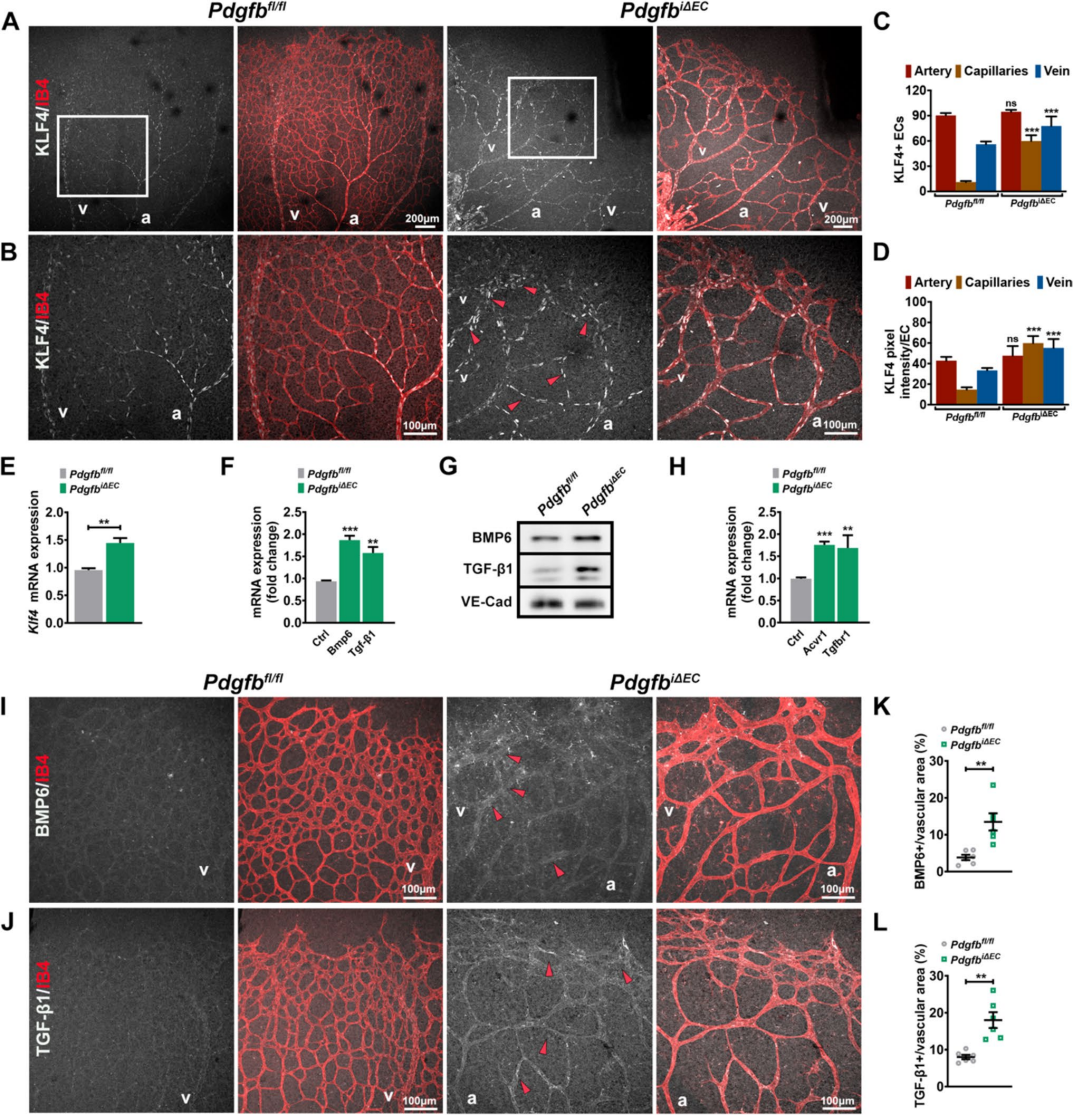
Capillary enlargement results in pathological flow-mediated Klf4 upregulation. **A**, **B**—small insets Co-labelling of P7 *Pdgfb*^*fl/fl*^ and *Pdgfb*^*iΔEC*^ retinas for KLF4 (white) and IB4 (red). Red arrowheads point towards KLF4 + ECs within the enlarged capillaries and in veins of *Pdgfb*^*iΔEC*^. **C**, **D** Quantification of KLF4 + ECs and of KLF4 labeling intensity per EC in arteries, capillaries and veins from *Pdgfb*^*fl/fl*^ and *Pdgfb*^*iΔEC*^ retinas (*Pdgfb*^*fl/fl*^ n = 6 *Pdgfb*^*i∆EC*^ n = 6 unpaired 2-tailed *t* test with Welch’s correction). **E**, **F** mRNA expression of *Klf4*, *Bmp6* and *Tgf-β1* in mLECs isolated from P7 *Pdgfb*^*fl/fl*^ and *Pdgfb*^*iΔEC*^ mice (*Pdgfb*^*fl/fl*^ n = 4/7 *Pdgfb*^*i∆EC*^ n = 4/7 unpaired 2-tailed *t* test with Welch’s correction). **G** WB for TGF-β1 and BMP6 in mLECs isolated from P7 *Pdgfb*^*fl/fl*^ n = 4 *Pdgfb*^*i∆EC*^ (**H**) *Acvr1 *and *Tgfrb1* mRNA expression in mLECs isolated from P7 *Pdgfb*^*fl/fl*^ and *Pdgfb*^*iΔEC*^ mice (*Pdgfb*^*fl/fl*^ n = 7, *Pdgfb*^*i∆EC*^ n = 7, unpaired 2-tailed *t* test). **I**, **J** Co-imunolabelling for IB4 (red) and BMP6 (white) (**I**) or TGF-Β1 (white) (**J**) of P7 *Pdgfb*^*fl/fl*^ and *Pdgfb*^*iΔEC*^ retinas. Red arrowheads point towards BMP6 + and TGF-Β1 + vessels. **K**, **L** Quantification of BMP6 (**K**) and TGF-β1 (**L**) immunolabelling intensity from P7 *Pdgfb*^*fl/fl*^ and *Pdgfb*^*iΔEC*^ retinas (*Pdgfb*^*fl/fl*^ n = 6, *Pdgfb*^*i∆EC*^ n = 6, unpaired 2-tailed *t* test with Welch’s correction). *ns* non-significant, **P < 0.01, ***P < 0.001. *a* artery, *v* vein

Capillary expansion upon *Pdgfb* loss results in an altered blood flow and/or an altered FSS within the capillaries. To visualize changes in flow patterns upon *Pdgfb* loss, we labelled retinas for KLF4, a mechanosensor previously identified as a robust readout of physiological FSS in the retina vasculature [21]. Whereas KLF4 is little expressed in low flow regions-vascular front and capillary ECs in control retinas, in *Pdgfb*^*iΔEC*^ retinas, KLF4 expression expanded throughout the capillary plexus with more KLF4 + ECs in the capillaries and veins engaged in the direct connections at the vascular front (Fig. 7A and B, quantified in C).

Interestingly, measuring KLF4 labeling intensity per single EC as a readout for its activation upon flow, KLF4 appeared more enhanced in capillaries and veins engaged in AV shunts in contrast to control retinas where KLF4 was intensely labeling the high flow arteries and the arterial branch points (Fig. 7D). Upregulation of *Klf4* expression was also validated by qPCR in *Pdgfb*^*iΔEC*^ mLECs (Fig. 7E). Together, these results emphasize that high KLF4 within the enlarged capillaries is likely a consequence of the combination of pathological flow and increased sensitivity of ECs to flow, thus supporting our in vitro data (Fig. 3K–L).

Interestingly, KLF4 was previously identified as a direct transcriptional activator of BMP6 [50], TGF-β1 [51] and Dll4 [52] ligands. As increased Dll4 and NOTCH activation was already confirmed (Fig. 4I, 6K), we performed qPCR and WB for *Tgf-β1* and *Bmp6* in *Pdgfb*^*fl/fl*^* and Pdgfb*^*iΔEC*^ mLECs. Both *Tgf-β1* and *Bmp6* mRNAs and proteins were upregulated upon *Pdgfb* loss (Fig. 7F, G). Interestingly, *Acvr1* and *Tgfbr1* coding for the Alk2 and Alk5 receptors were also upregulated (Fig. 7H).To validate these findings, we next labelled retinas for Bmp6 (Fig. 7I) and Tgf-β1 (Fig. 7J). While, the ligands were very little expressed in control retinas, both BMP6 and TGF-β1 showed increased expression at the vascular front and in the AV shunts (Fig. 7I–L). Thus, within the dilated capillaries, endothelial overactivation of KLF4 is associated with TGF-β1 and BMP6 upregulation that presumably mediates the downstream activation of canonical SMADs simultaneously with increased Dll4-mediated NOTCH activation.

## Discussion

MCs and ECs act in concert to regulate angiogenesis and to maintain vascular homeostasis [2]. On the one hand, angiocrine factors produced by the endothelium promote PC recruitment and thereafter their maintenance on the endothelial layer. In turn, MCs provide dynamic control of blood perfusion within the vascular networks and thus, maintain an appropriate vascular tone. Yet, despite recent advances, how dysfunctional ECs-MCs crosstalk contributes to the progression of many diseases, including high-flow mediated-AVM pathogenesis, still remains poorly understood. AVMs are direct connections between an artery and a vein with a loss of capillary bed. Sporadic or inherited, these vascular lesions show a localized drop of PCs. Whether PC loss from developing capillaries triggers AVM formation or it is secondary to pathological hemodynamics-mediated AVMs remains an open question in the field. Furthermore, which angiocrine-paracrine signals are essential for maintaining functional PCs to stabilize the developing endothelium remain largely unknown.

Recent advances in the field identified key signaling components in MCs required to protect the endothelium against forming AVMs. One such example is NOTCH signaling which acts upstream of PDGFRβ to regulate PC survival and proliferation [22]. Downstream of PDGFRβ, ablation of *Srf* transcription factor also leads to AVM formation due to inadequate PC migration towards the endothelium and contractile SMC-mediated loss of vascular tone [24].

On the other hand, gain of function of NOTCH [23] or LOF of canonical BMP9/10 signaling in ECs leads to AVM formation [3, 19–21], and both pathways were shown to regulate *Pdgfb* expression [3, 53]. Interestingly the two pathways converge in regulating many common downstream genes [54], including N-cadherin, a critical player in mediating EC-PC communications, downstream of PDGFB-PDGFRβ signaling [55]. Physiological flow regulates NOCTH activation in ECs [56] and BMP9 is indispensable for flow-induced expression of master regulators of MC recruitment in ECs, including PDGFB [3], thus emphasizing intertwined upstream regulatory mechanisms of angiocrine PDGFB production to maintain vascular homeostasis.

Yet, disrupting PDGFB/PDGFRβ signaling either in endothelium or MCs leads to a plethora of vascular defects, but this angiocrine-paracrine signaling axis has never been interrogated in the context of inducing AVM pathogenesis. Interestingly, overexpression of PDGFB reduces hemorrhage in bAVMs and HHT patients [18, 57]. Yet, to our knowledge, our study is the first to provide a genetic link between *Pdgfb* ablation-mediated PC loss and AVM development in mice.

Herein, employing inducible model of *Pdgfb* ablation, we demonstrate that EC-derived *Pdgfb* is indispensable for PC recruitment, but also for PC maintenance on developing vessels. Depletion of *Pdgfb* either before or after the vasculature differentiates, results in PC loss and capillary enlargement across multiple vascular beds and AV shunting.

Yet, the resulting AVM-like structures are not identical to the inherited HHT-like AVMs. Upon *Pdgfb* ablation, AVMs-like structures form at the vascular front in low flow regions, suggesting distinct cell events whose disruption initiate and contribute to these lesions. Indeed, the resulting AVMs-like structures are characterized by enlarged capillaries containing non-proliferative, hyperplastic and hypertrophic ECs with un-organized junctions. Similarly to *Pdgfb*^*ret/ret*^ brain capillaries [35], retinal capillary ECs in *Pdgfb*^*iΔEC*^ showed a venous skewing, emphasizing a conserved role for PDGFB signaling in maintaining capillary zonation. Yet, specifically and exclusively, the AV shunts displayed gain of arterial identity with some arterial ECs present in the veins, emphasizing either an arterial origin, or disruption in flow-migration coupling as the triggering event for capillary enlargement. While we failed to find an arterial origin for the AVMs-like structures upon *Pdgfb* loss, instead similarly to *Alk1* or *Eng* depletion [30, 31, 58], we identified an altered EC migration among the hallmarks that could precipitate AV shunting. This underscores an important role for capillary and/or venous PDGFB-PDGFRβ signaling in flow-migration coupling.

If the perturbed migration is a consequence of defective hemodynamics within the hyperplastic and hypertrophic capillaries or it is due to altered attraction-repulsive mechanisms that further precipitate AV shunting, remains to be further investigated. Indeed, *Pdgfb* ablation resulted in activation of the EphrinB2-EphB4 signaling mediated arterial-venous EC repulsive-attraction [59] and interestingly also of Apelin/Apj signaling pathway. Yet, loss of Apelin, a BMP suppressed target gene [46], leads to a defective EC migration against the bloodstream [60]. One possible explanation for the discrepant results is that increased *Apelin* expression upon *Pdgfb* loss is mediated through another upstream regulator, such as NOTCH [60].

Abundant KLF4 within the enlarged capillaries engaged in AV shunts indicates perturbed hemodynamics with a disrupted vascular tone. Yet, it may also suggest an increased sensitivity of ECs to FSS upon *Pdgfb* loss, as recently reported in AVMs upon *Smad4* depletion [61]. Interestingly, hypertensive (αSMA positive) veins at the expense of hypotensive arteries (αSMA negative) seen in the *Pdgfb* LOF retinas and in human AVMs [17], further confirm an altered hemodynamics. Surprisingly, in vitro we could show that *PDGFB* knock down potently augments flow-induced morphological events. Whether PDGFB cell autonomously restrains FSS-mediated EC responses to maintain capillary EC size, caliber and zonation by restricting KLF4-mediated hemodynamic changes or these cell events occur due to ensheathing PCs-mediated intrinsic and/or extrinsic signaling is so far unclear. At this point, we can only speculate that vascular lesions observed in these mutant mice are rather secondary effects of PC coverage defects. Yet, the possibility for an autocrine contribution to AVM pathogenesis cannot fully be excluded. Whether the venous or capillary PDGFB protects the endothelium against AVMs also needs to be investigated further and will require generation of capillary- or vein-specific Cre driver lines.

Based on our findings, we propose a model in which EC *Pdgfb* ablation*-*mediated loss of PCs results in capillary enlargement leading to perturbed hemodynamics and an increase of EC sensitivity to flow, which will result in KLF4 overactivation. Excessive KLF4 then triggers changes in arterial-venous identity regulatory programs: activation of NOTCH and TGF-β1-Smad3 mediated arterial identity in the same time with BMP6-mediated venous identity through Smad1/5 activation. This altered EC fate pattern together with loss of repulsion-attraction regulatory mechanisms then triggers a confusion in the migration of ECs against the bloodstream that leads to an accumulation of ECs in capillaries, further increasing capillary caliber and ultimately giving rise to AVMs.

## Materials and methods

### Animal experiments

Deletion of endothelial *Pdgfb* (*Pdgfb*^*iΔEC*^) was achieved by crossing *Pdgfb fl/fl* with Tx inducible *Cdh5-Cre*^*ERT2*^ mice. Deletion of *Pdgfb* in the arterial ECs (*Pdgfb*^*iΔBMX*^) was achieved by crossing *Pdgfb fl/fl* to the Tx inducible *Bmx*-Cre^ERT2^;mTmG mice. Gene deletion was achieved by i.p injections of 100 µg Tx (Sigma, T5648) into *Pdgfb*^*iΔEC*^ or *Pdgfb*^*iΔBMX*^ at postnatal days (P0–P2) or (P5–P7). Tx-injected Cre-negative littermates (*fl/fl*) were used as controls. Mice were maintained under standard specific pathogen-free conditions, and animal procedures were approved by the animal welfare commission of the Regierungspräsidium Karlsruhe (Karlsruhe, Germany) and The Comité Institutionnel des Bonnes Pratiques Animales en Recherche (CIBPAR), Canada.

### Latex red-dye injection

P7 or P12 pups were anaesthetized and perfused with 2 ml of PBS. Latex dye (Connecticut Valley Biological Supply Company) was slowly injected through left ventricle with an 1 ml insulin syringe. To visualize pulmonary arteries, latex was injected into the right ventricle. Tissues were fixed in 4% PFA at 4 °C overnight and washed in PBS the following day. The dissected organs were imaged under a dissection microscope.

### Reagents and antibodies

For immunodetection: anti-NG2 (#AB5320, 1:200, millipore), Isolectin B4 (IB4, #121412, 10 μg/ml, Life Technologies), anti-GOLPH4 (#ab28049; 1:200, Abcam), anti-ERG (#92513; 1:200, Abcam), anti-KLF4 (#AF3158, 1:200, R&D systems), anti-SOX17 (#AF1924, 1:200, R&D systems), anti-VE-cadherin (#555289, 1:400, BD), anti-Jag1 (#AF599, 1:200, R&D systems), anti-Endomucin (#sc-65495, 1:200, Santa Cruz), anti-Dll4 (#AF1389, 1:200, R&D systems), anti-phospho-SMAD3 (#ab52903, 1:100, Abcam), anti-phospho-SMAD1/5 (#13820S, 1:100, Cell Signalling), anti-ID1 (#AF4377, 1:100, R&D systems), anti-Endoglin (#AF1320, 1:100, R&D systems), anti-GM130 (#610823; 1:600 BD Bioscience), anti-BMP6 (#ab15640, 1:100, Abcam), anti-TGF-β1 (#MAB7666, 1:100, R&D systems), anti-αSMA (#ab184675,1:600, Abcam), anti-ICAM2 (#553326, 1:200, BD), anti-Collagen IV (#ab6586, 1:200, Abcam) and anti-P21 from CNIO-Centro Nacional de Investigaciones Oncológicas).

For WB: anti-PPDGFB (#ab23914, 1:1,000, Abcam), anti-SMAD1 (#6944S, 1:1,000, Cell Signalling), anti-SMAD2/3 (#8685S; 1:1000; Cell Signalling), anti-phospho-SMAD1/5/8 (#13820S, 1:1000, Cell Signalling), anti-phospho-SMAD3 (#ab52903, 1:1000, Abcam), anti-TGF-β1 (#sc52893, 1:500, Santa cruz) and anti-VE-cadherin (#sc9989, 1:200, Santa cruz).

Appropriate secondary antibodies were fluorescently labelled (Alexa Fluor donkey anti-rabbit, #R37118, Alexa Fluor donkey anti-goat 555, #A-21432, 1:250, Thermo Fisher) or conjugated to horseradish peroxidase for WB (Anti-Rabbit #PI-1000-1 and Anti-mouse #PI-2000-1 IgG (H + L), 1:5000, Vector Laboratories).

### Proliferation assay in vivo

For analysis of cell proliferation in the retinas, pups were injected i.p with 5-ethynyl-2-deoxyuridine (EdU, 100 mg/Kg; Thermo Fischer Scientific) 4 h before dissection. Retinas were collected and EdU labelling was detected with the Click-it EdU Alexa Fluor-488 Imaging Kit (C10337, Life Technologies) according to the manufacturer’s instructions.

### Isolation of mLECs

Mouse lung ECs were isolated using MACS (Miltenyi Biotec). Mice were sacrificed and lungs were harvested immediately. Lungs were cut into small pieces and digested with collagenase I at 37 °C for 45 min. Tissue suspension was passed through 70 μm cell strainer, incubated with red blood cell lysis Buffer (11814389001, Sigma) and washed several times with PEB buffer (0.5% BSA, 2 mM EDTA in PBS). Cell suspension was incubated with CD45 MicroBeads (1:10) for 15 min at 4 °C and passed through the MS columns. The unlabeled cells were collected and centrifuged at 1000 rpm at 4 °C for 10 min. Cell pellets was resuspended with PEB buffer and incubated with CD31 MicroBeads (1:10) for 15 min at 4 °C and applied onto MS columns, eluted from the columns with PEB buffer and directly used for RNA or protein extraction.

### Quantitative PCR

RNA extraction from mLECs were performed using RNeasy-kit (74106, Qiagen) according to the manufacturer’s instructions. The RNA was reverse transcribed using High-Capacity cDNA Reverse Transcription Kit (4368813, Thermo Fisher) and quantitative PCR assays were carried out using PowerUP SYBR Green Master Mix (A25778, Thermo Fisher) with a QuantStudio 3 (Thermo Fisher) according to the manufactures protocol. The following primers were used for mLECs: *Eng* (Forward: AGGGGTGAGGTGACGTTTAC, Reverse: GTGCCATTTTGCTTGGATGC), *Thbs1* (Forward: CCTGCCAGGGAAGCAACAA, Reverse: ACAGTCTATGTAGAGTTGAGCCC), *Fn1* (Forward: ATGTGGACCCCTCCTGATAGT, Reverse: GCCCAGTGATTTCAGCAAAGG), *Dll4* (Forward: TTCCAGGCAACCTTCTCCGA, Reverse: ACTGCCGCTATTCTTGTCCC), *Notch1* (Forward: GATGGCCTCAATGGGTACAAG, Reverse: TCGTTGTTGTTGATGTCACAGT), *Notch4* (Forward: GAACGCGACATCAACGAGTG, Reverse: GGAACCCAAGGTGTTATGGCA), *Hey1* (Forward: CCGACGAGACCGAATCAATAAC, Reverse: TCAGGTGATCCACAGTCATCTG), *Hey2* (Forward: CGCCCTTGTGAGGAAACGA, Reverse: CCCAGGGTAATTGTTCTCGCT), *Hes1* (Forward: TCAACACGACACCGGACAAAC, Reverse: ATGCCGGGAGCTATCTTTCTT), *Klf4* (Forward: GGCGAGTCTGACATGGCTG, Reverse: GCTGGACGCAGTGTCTTCTC), *Bmp6* (Forward: GCGGGAGATGCAAAAGGAGAT, Reverse: ATTGGACAGGGCGTTGTAGAG), *Tgf-β1* (Forward: CCACCTGCAAGACCATCGAC, Reverse: CTGGCGAGCCTTAGTTTGGAC), *Pdgfb* (Forward: CATCCGCTCCTTTGATGATCTT, Reverse: GTGCTCGGGTCATGTTCAAGT), *Ephb4* (Forward: GGAAACGGCGGATCTGAAATG, Reverse: TGGACGCTTCATGTCGCAC), *Nrp2* (Forward: GCTGGCTACATCACTTCCCC, Reverse: GGGCGTAGACAATCCACTCA), *Nr2f2* (Forward: CATCGAGAACATTTGCGAACTG, Reverse: GTCGGCTGACATGGGTGAAG), *Aplnr* (Forward: CCAGTCTGAATGCGACTACG, Reverse: CTCCCGGTAGGTATAAGTGGC), *Sox17* (Forward: GATGCGGGATACGCCAGTG, Reverse: CCACCTCGCCTTTCACCTTTA), *Nrp1* (Forward: ACCTCACATCTCCCGGTTACC, Reverse: AAGGTGCAATCTTCCCACAGA), *Unc5b* (Forward: CGGGACGCTACTTGACTCC, Reverse: GGTGGCTTTTAGGGTCGTTTAG), *Efnb2* (Forward: TTGCCCCAAAGTGGACTCTAA, Reverse: GCAGCGGGGTATTCTCCTTC), *Tgfbr1* (Forward: AAAACAGGGGCAGTTACTACAAC, Reverse: TGGCAGATATAGACCATCAGCA), *Acvr1* (Forward: ATGGTCGATGGAGTAATGATCCT, Reverse: TGCTCATAAACCTGAAAGCAGC).

### Retina isolation and immunostaining

The eyes from P7 or P12 pups were fixed in 4% PFA for 17 min at room temperature (rt). Post dissection, the retinas were washed 3 times with PBS and then incubated in blocking buffer (1% fetal bovine serum, 3% BSA, 0.5% Triton X-100, 0.01% sodium deoxycholate, 0.02% sodium azide in PBS at pH 7.4) for 15 min at rt. Post-blocking, retinas were incubated with specific antibodies diluted in blocking buffer overnight, at 4 °C. The next day, retinas were washed and incubated with anti-IB4 together with the corresponding secondary antibody in PBLEC buffer (1 mM CaCl_2_, 1 mM MgCl_2_, 1 mM MnCl_2_ and 0,25% Triton X-100 in PBS) for 1 h at rt, postfixed for 20 min with 4% PFA in rt, washed and mounted in fluorescent mounting medium (RotiMount FluorCare #HP19.1, CarlRoth). Whole-mount retina images were acquired using Zeiss LSM800 confocal microscope with Airyscan Detector and the Zeiss ZEN software. Quantification of retinal vasculature was analyzed using Fiji.

### Western blotting

Total protein from the mLECS were lysed with Laemmli buffer (1610747, Biorad). Samples were separated on 10% SDS-PAGE gels and transferred on 0.2 µm nitrocellulose membranes (10600004, GE Healthcare). Western blots were developed with the Clarity Western ECL Substrate (1705061, Biorad) on a Luminescent image Analyzer, Fusion FX (Vilber). Bands’ intensity were quantified using ImageJ.

### Cell culture, siRNA transfection

Human umbilical vein endothelial cells (HUVECs), from Lonza (#C2519A), were grown in culture using Endothelial Cell Growth Medium MV2 supplemented with a mix of additives (#C-22022, PromoCell) and 1% Penicillin/Streptomycin solution (#P4333, Sigma-Aldrich). These cells were cultured in an incubator set at 37 °C, with a 5% CO2 atmosphere and 100% humidity. Deletion of *PDGFB* was carried out by transfecting 25 pmol of PDGFB siRNA (ON-TARGETplus Human PDGFB siRNA Smart Pool, #L-011749-00-0005) using Lipofectamine RNAiMax (Invitrogen) in OPTI-MEM. The experiments were conducted within the window of 48 to 72 h post-transfection, and the results were compared to HUVECs transfected with siRNA *CTRL* (ON-TARGETplus Non-Targeting Pool D-001810-10-05).

### Exposure of ECs to FSS

siRNA tansfected HUVECs were placed onto a µ-Slide VI^0.4^ (Ibidi, #80601) and exposed to laminar fluid shear stress levels of 1 and 12 DYNES/cm^2^ for 24 h using the Ibidi pump system (Ibidi, #10902).

### Image analysis

PC and αSMA coverage were assessed by quantifying the area of NG2-positive or αSMA positive area normalised to the area of IB4-positive vasculature. The vascular area was quantified by determining the threshold value of EC area relative to the total field area. The radial length was determined by measuring the distance from the optic nerve to the outer edge of the vascular front for each leaflet. The vessel diameter was calculated by averaging of six-eight measurements per retina. The number of side branches (intersections in major vessels) was calculated in 3–4 major vessels per retina. The number of tip cells in sprouting vascular fronts was measured in four pictures per retina.

### Statistical analysis

All data are presented as mean ± standard error of the mean (SEM). Samples with equal variances were tested using Mann–Whitney *U* test or two-tailed Student’s *t* test between groups using GraphPad Prism (GraphPad Software). P value < 0.05 was considered to be statistically significant. Statistical analyses were performed for all quantitative data using Prism 9.0 (Graph Pad).

### Supplementary Information

Below is the link to the electronic supplementary material.

Supplementary file1 (TIF 5610 KB)
Suppl. Figure 1. Loss of *Pdgfb* in ECs impairs retinal vascular development. **a**, **b** *Pdgfb* mRNA and protein expression by qPCR (**a**) and WB, respectively (**b**) in purified mouse lung endothelial cells (mLECs) from P7 TX induced *Pdgfb*^*fl/fl*^ and *Pdgfb*^*i*∆*EC*^ neonates (*Pdgfb*^*fl/fl*^ n=6, *Pdgfb*^*i*∆*EC*^ n=6, unpaired 2-tailed t test). **c** Body weight of P7 TX induced *Pdgfb*^*fl/fl*^ and *Pdgfb*^*i*∆*EC*^ mice (*Pdgfb*^*fl/fl*^ n=16, *Pdgfb*^*i*∆*EC*^ n=20, unpaired 2-tailed t test). **d** Quantification of the retinal vascular area (*Pdgfb*^*fl/fl*^ n=9, *Pdgfb*^*i*∆*EC*^ n=10, unpaired 2-tailed t test), radial length (*Pdgfb*^*fl/fl*^ n=8, *Pdgfb*^*i*∆*EC*^ n=10, unpaired 2-tailed t test), number of side branches (*Pdgfb*^*fl/fl*^ n=12, *Pdgfb*^*i*∆*EC*^ n=12, Mann-Whitney U-test), and vessel diameter in *Pdgfb*^*fl/fl*^ and *Pdgfb*^*i*∆*EC*^ retinas (*Pdgfb*^*fl/fl*^ n=12, *Pdgfb*^*i*∆*EC*^ n=14, unpaired 2-tailed t test). **e** High-magnification images of the angiogenic growth front in *Pdgfb*^*fl/fl*^ and *Pdgfb*^*i*∆*EC*^ retinas. Red arrowheads indicate tip ECs. **f** Quantification of the number of tip cells (*Pdgfb*^*fl/fl*^ n=30, *Pdgfb*^*i*∆*EC*^ n=30, unpaired 2-tailed t test), number of filopodia per tip cell (*Pdgfb*^*fl/fl*^ n=20, *Pdgfb*^*i*∆*EC*^ n=20, unpaired 2-tailed t test) and the filopodia length (*Pdgfb*^*fl/fl*^ n=30, *Pdgfb*^*i*∆*EC*^ n=30, unpaired 2-tailed t test). *ns* non-significant, **P<0.01, ***P<0.001. *a* artery, *v* vein

Supplementary file2 (TIF 64999 KB)
Suppl. Figure 2. Loss of *Pdgfb* leads to aberrant SMC coverage, capillary enlargement, vessel regression and cell cycle arrest. **a**, **c**, **e**, **g** Co-immunostaining with αSMA (white) and IB4 (red) (**a**), ICAM2 (green) and IB4 (blue) (**c**), ColIV (red) and IB4 (green) (**e**) P21 (green), IB4 (blue) and ERG (red) (**g**) of P7 *Pdgfb*^*fl/fl*^ and *Pdgfb*^*i*∆*EC*^ mouse retinas. Green arrowheads in a point towards αSMA+ cells in capillaries and veins. Higher magnification of insets in a emphasizing loss of αSMA in arteries from *Pdgfb*^*i*∆*EC*^. Yellow arrowheads in c point towards capillaries with an increased lumen. White arrowheads in e point towards ColIV+/IB4- capillaries. Yellow arrowheads in g point towards AV shunts. **b**, **d**, **f**, **h** Quantification of percentage of αSMA coverage (*Pdgfb*^*fl/fl*^ n=4, *Pdgfb*^*i*∆*EC*^ n=8, unpaired 2-tailed t test with Welch’s correction) (**b**), of Icam2+/IB4+ area (*Pdgfb*^*fl/fl*^ n=6, *Pdgfb*^*i*∆*EC*^ n=6, unpaired 2-tailed t test) (**d)** of the number of empty sleeves (*Pdgfb*^*fl/fl*^ n=6, *Pdgfb*^*i*∆*EC*^ n=6, unpaired 2-tailed t test) (**f**), of p21+ERG+ per field of view (*Pdgfb*^*fl/fl*^ n=4, *Pdgfb*^*i*∆*EC*^ n=10, unpaired 2-tailed t test with Welch’s correction) (**h**) in P7 *Pdgfb*^*fl/fl*^ and *Pdgfb*^*i*∆*EC*^ mouse retinas. *P<0.05, ***P<0.001. *a* artery, *v* vein
