# Supplementary figures and images for: Role of endothelial PDGFB in arterio-venous malformations pathogenesis
Source: Angiogenesis. 2023 Dec 9;27(2):193–209. doi: 10.1007/s10456-023-09900-w (PMC11021264; doi:10.1007/s10456-023-09900-w)

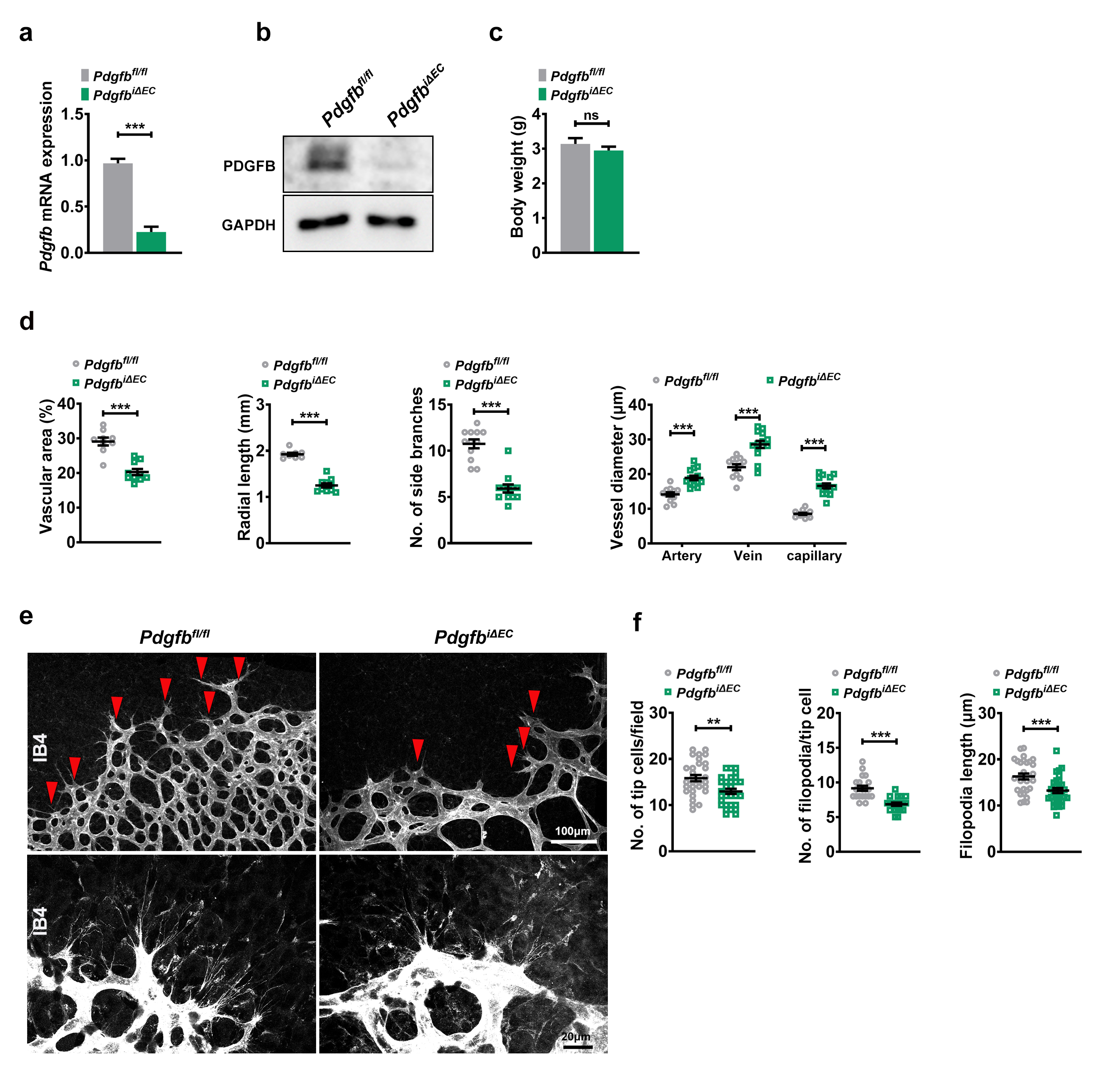

Supplement: Supplementary file 1 — Supplementary file1 (TIF 5610 KB) Suppl. Figure 1. Loss of Pdgfb in ECs impairs retinal vascular development. a, b Pdgfb mRNA and protein expression by qPCR (a) and WB, respectively (b) in purified mouse lung endothelial cells (mLECs) from P7 TX induced Pdgfbfl/fl and Pdgfbi∆EC neonates (Pdgfbfl/fl n=6, Pdgfbi∆EC n=6, unpaired 2-tailed t test). c Body weight of P7 TX induced Pdgfbfl/fl and Pdgfbi∆EC mice (Pdgfbfl/fl n=16, Pdgfbi∆EC n=20, unpaired 2-tailed t test). d Quantification of the retinal vascular area (Pdgfbfl/fl n=9, Pdgfbi∆EC n=10, unpaired 2-tailed t test), radial length (Pdgfbfl/fl n=8, Pdgfbi∆EC n=10, unpaired 2-tailed t test), number of side branches (Pdgfbfl/fl n=12, Pdgfbi∆EC n=12, Mann-Whitney U-test), and vessel diameter in Pdgfbfl/fl and Pdgfbi∆EC retinas (Pdgfbfl/fl n=12, Pdgfbi∆EC n=14, unpaired 2-tailed t test). e High-magnification images of the angiogenic growth front in Pdgfbfl/fl and Pdgfbi∆EC retinas. Red arrowheads indicate tip ECs. f Quantification of the number of tip cells (Pdgfbfl/fl n=30, Pdgfbi∆EC n=30, unpaired 2-tailed t test), number of filopodia per tip cell (Pdgfbfl/fl n=20, Pdgfbi∆EC n=20, unpaired 2-tailed t test) and the filopodia length (Pdgfbfl/fl n=30, Pdgfbi∆EC n=30, unpaired 2-tailed t test). ns non-significant, **P<0.01, ***P<0.001. a artery, v vein [file 10456_2023_9900_MOESM1_ESM.tif]

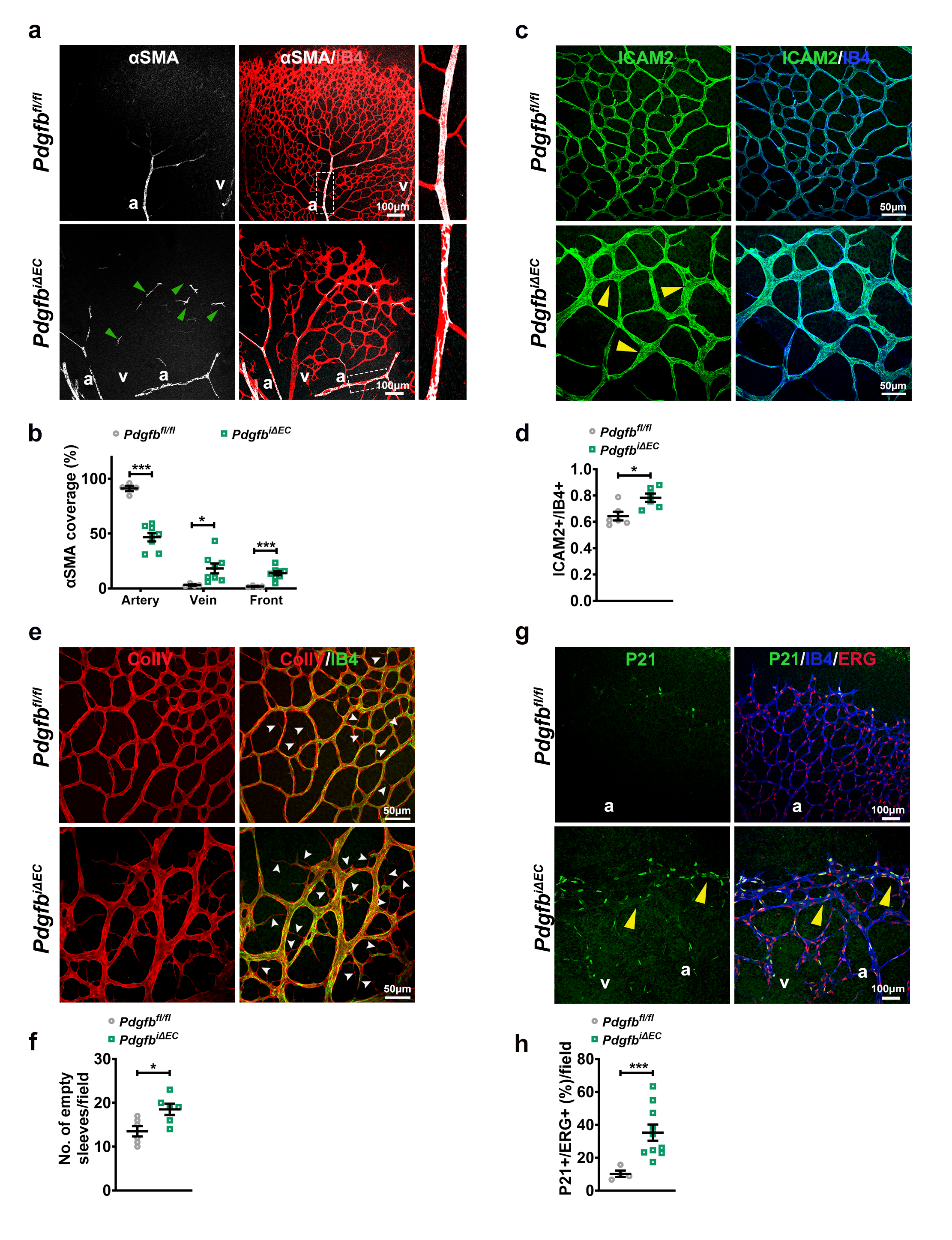

Supplement: Supplementary file 2 — Supplementary file2 (TIF 64999 KB) Suppl. Figure 2. Loss of Pdgfb leads to aberrant SMC coverage, capillary enlargement, vessel regression and cell cycle arrest. a, c, e, g Co-immunostaining with αSMA (white) and IB4 (red) (a), ICAM2 (green) and IB4 (blue) (c), ColIV (red) and IB4 (green) (e) P21 (green), IB4 (blue) and ERG (red) (g) of P7 Pdgfbfl/fl and Pdgfbi∆EC mouse retinas. Green arrowheads in a point towards αSMA+ cells in capillaries and veins. Higher magnification of insets in a emphasizing loss of αSMA in arteries from Pdgfbi∆EC. Yellow arrowheads in c point towards capillaries with an increased lumen. White arrowheads in e point towards ColIV+/IB4- capillaries. Yellow arrowheads in g point towards AV shunts. b, d, f, h Quantification of percentage of αSMA coverage (Pdgfbfl/fl n=4, Pdgfbi∆EC n=8, unpaired 2-tailed t test with Welch’s correction) (b), of Icam2+/IB4+ area (Pdgfbfl/fl n=6, Pdgfbi∆EC n=6, unpaired 2-tailed t test) (d) of the number of empty sleeves (Pdgfbfl/fl n=6, Pdgfbi∆EC n=6, unpaired 2-tailed t test) (f), of p21+ERG+ per field of view (Pdgfbfl/fl n=4, Pdgfbi∆EC n=10, unpaired 2-tailed t test with Welch’s correction) (h) in P7 Pdgfbfl/fl and Pdgfbi∆EC mouse retinas. *P<0.05, ***P<0.001. a artery, v vein [file 10456_2023_9900_MOESM2_ESM.tif]
